# The PD-1: PD-L1 pathway promotes development of brain-resident memory T cells following acute viral encephalitis

**DOI:** 10.1186/s12974-017-0860-3

**Published:** 2017-04-13

**Authors:** Sujata Prasad, Shuxian Hu, Wen S. Sheng, Priyanka Chauhan, Amar Singh, James R. Lokensgard

**Affiliations:** Department of Medicine, Neurovirology Laboratory, University of Minnesota, 3-107 Microbiology Research Facility, 689 23rd Avenue S.E., Minneapolis, MN 55455 USA

## Abstract

**Background:**

Previous work from our laboratory has demonstrated that during acute viral brain infection, glial cells modulate antiviral T cell effector responses through the PD-1: PD-L1 pathway, thereby limiting the deleterious consequences of unrestrained neuroinflammation. Here, we evaluated the PD-1: PD-L1 pathway in development of brain-resident memory T cells (bT_RM_) following murine cytomegalovirus (MCMV) infection.

**Methods:**

Flow cytometric analysis of immune cells was performed at 7, 14, and 30 days post-infection (dpi) to assess the shift of brain-infiltrating CD8^+^ T cell populations from short-lived effector cells (SLEC) to memory precursor effector cells (MPEC), as well as generation of bT_RMs_.

**Results:**

In wild-type (WT) animals, we observed a switch in the phenotype of brain-infiltrating CD8^+^ T cell populations from KLRG1^+^ CD127^−^ (SLEC) to KLRG1^−^ CD127^+^ (MPEC) during transition from acute through chronic phases of infection. At 14 and 30 dpi, the majority of CD8^+^ T cells expressed CD127, a marker of memory cells. In contrast, fewer CD8^+^ T cells expressed CD127 within brains of infected, PD-L1 knockout (KO) animals. Notably, in WT mice, a large population of CD8^+^ T cells was phenotyped as CD103^+^ CD69^+^, markers of bT_RM_, and differences were observed in the numbers of these cells when compared to PD-L1 KOs. Immunohistochemical studies revealed that brain-resident CD103^+^ bT_RM_ cells were localized to the parenchyma. Higher frequencies of CXCR3 were also observed among WT animals in contrast to PD-L1 KOs.

**Conclusions:**

Taken together, our results indicate that bT_RMs_ are present within the CNS following viral infection and the PD-1: PD-L1 pathway plays a role in the generation of this brain-resident population.

**Electronic supplementary material:**

The online version of this article (doi:10.1186/s12974-017-0860-3) contains supplementary material, which is available to authorized users.

## Background

Infection of the central nervous system (CNS) presents unique challenges to effective pathogen control, as brain infection may rapidly progress causing substantial damage or even death. Neuroimmune responses are critical for antiviral defense, but extensive damage to this generally non-regenerating tissue must be avoided [1]. It is well established that different immune mechanisms are very specifically tailored to control infections in particular organs. Recent studies have demonstrated that after clearance of many acute viral infections, CD8^+^ T lymphocytes generate a population of long-lived, non-recirculating tissue-resident memory cells (T_RM_) in non-lymphoid tissue; and it is becoming increasingly clear that these T_RM_ cells play critical roles in controlling re-encountered infection and accelerating the process of pathogen clearance [2–5].

The CNS can be a target of acute viral infection, as well as a reservoir of latent and persistent virus. During acute viral infection, most pathogens are rapidly cleared through the generation of a large number of short-lived effector T cells (SLEC). Simultaneously, the T cell response is triggered to generate a subset identified as memory precursor effector cells (MPEC). These MPEC begin to develop into a tissue-resident memory (T_RM_) phenotype shortly after infection. Recent work by several groups provides evidence that there is a clear distinction between terminal effector and memory cells based on heterogeneity in expression of killer cell lectin-like receptor G1 (KLRG1) [6–8]. We have recently characterized brain-infiltrating T cells which persist within the tissue after acute murine cytomegalovirus (MCMV) infection. We showed that infiltrating CD8^+^ T cell populations shift from SLEC to clear infection to MPEC that protect against re-challenge. The shift of prominent SLEC populations to MPEC populations is concomitant with transition from acute through chronic phases of infection. In addition, these cells were found to selectively express the integrin CD103, a marker of brain T_RM_ (bT_RM)_ cells and persist long-term within the CNS [9].

Resolution of adaptive immune responses and generation of immunological memory is an essential process to confer long-term protective immunity particularly in immune-privileged tissue-like brain. Inflammation within different anatomical sites of brain dramatically increases the infiltration and migration of lymphocytes and effector molecules. We understand much about the infiltrating T cell mediated immune response and the penetration of T cells within the infected brain parenchyma [10]. However, better understanding of the association between inflammation and the establishment of T_RM_ will inform us about the protective effects of neuroimmune responses to re-infection or viral reactivation.

T_RM_ cells are characterized by their non-recirculating, resident nature in tissues. It is well reported that T_RM_ cells often express α_E_β_7_. α_E_, otherwise known as CD103, has been identified as a marker of particular types of T_RM_ cells. High expression of CD103 and CD69 is a common feature of resident memory cells observed in epithelial tissue [11, 12]. Whereas, effector and resident memory cells in circulation appear to lack expression of both CD103 and CD69 [13, 14]. It has been shown that CD69 expression is required for the optimal formation of T_RM_ following herpes simplex virus (HSV) infection in tissues such as the skin and dorsal root ganglia [2, 15]. In addition, experiments using the skin, lung, and gut show differential expression of CCR7, as well as CXCR3, which define the migration properties of T cells [16–18]. However, further insight into factors responsible for development of T_RM_ is required. Given the importance of the formation of brain (bT_RM_) cells, there is surprisingly little known about how glial cells contribute to their formation.

The programmed death receptor-1 (PD-1): programmed death ligand-1 (PD-L1) pathway is central in controlling interactions between host defense and invading pathogens. Accumulating evidence suggests that during neuroinflammation, PD-L1 expression is increased on microglial cells, as well as astrocytes [19]. These findings suggest that resident glial cells limit CNS pathology through suppression of proinflammatory cytokine production from brain-infiltrating T cells via activation of the PD-1: PD-L1 pathway [20]. PD-L1 expression on glial cells has also been shown to limit immune-mediated tissue damage in models of multiple sclerosis, as well as during acute viral encephalitis [19, 21, 22].

We have previously investigated the role of PD-1: PD-L1 signaling in regulating immunopathology through functional inhibition of effector CD8^+^ T cells within the post-encephalitic brain following MCMV infection [19]. In the present study, we investigated the involvement of PD-1: PD-L1 signaling in the retention of CD8^+^-gated CD103^+^CD69^+^ T cells and the development of bT_RM_. Using our murine model of MCMV infection, we performed phenotypic analysis of CD8^+^ lymphocytes residing within the chronically infected brain to characterize bT_RM_. We also compared their development in wild-type (WT) animals to that in PD1- and PD-L1 knockout mice.

## Methods

### Ethical statement

This study was carried out in strict accordance with recommendations in the Guide for the Care and Use of Laboratory Animals of the National Institutes of Health. The protocol was approved by the Institutional Animal Care and Use Committee (Protocol Number: 1402-31338A) of the University of Minnesota. All surgery was performed under ketamine/xylazine anesthesia and all efforts were made to minimize suffering.

### Virus and animals

RM461, a MCMV expressing *Escherichia coli* β-galactosidase under the control of the human ie1/ie2 promoter/enhancer [23], was kindly provided by Edward S. Mocarski. The virus was maintained by passage in weanling female Balb/c mice. Salivary gland-passed virus was then grown in NIH 3T3 cells for two passages, which minimized any carry-over of salivary gland tissue. Infected 3T3 cultures were harvested at 80 to 100% cytopathic effect and subjected to three freeze–thaw cycles. Cellular debris was removed by centrifugation (1000×*g*) at 4 °C, and the virus was pelleted through a 35% sucrose cushion (in Tris-buffered saline [50 mM Tris–HCl, 150 mM NaCl, pH 7.4]) at 23,000×*g* for 2 h at 4 °C. The pellet was suspended in Tris-buffered saline containing 10% heat-inactivated fetal bovine serum (FBS). Viral stock titers were determined on 3T3 cells as 50% tissue culture infective doses (TCID_50_) per milliliter. Six to eight weeks old C57B/6 mice were obtained from Charles River Laboratories (Wilmington, MA), while PD-L1 KO and PD-1 KO animals were kindly provided by Arlene Sharpe (Harvard University) and Sing Sing Way (Cincinnati Children’s Hospital, Cincinnati, OH), respectively.

### Intracerebroventricular infection of mice

Infection of mice with MCMV was performed as previously described [24]. Briefly, female mice (6–8 weeks old) were anesthetized using a combination of ketamine and xylazine (100 mg and 10 mg/kg body weight, respectively) and immobilized on a small animal stereotactic instrument equipped with a Cunningham mouse adapter (Stoelting Co., Wood Dale, IL). The skin and underlying connective tissue were reflected to expose reference sutures (sagittal and coronal) on the skull. The sagittal plane was adjusted such that the bregma and lambda were positioned at the same coordinates on the vertical plane. Virulent, salivary gland-passaged MCMV RM461 (1 × 10 [5] TCID_50_ units in 10 μL), was injected into the right lateral ventricle at 0.9 mm lateral, 0.5 mm caudal, and 3.0 mm ventral to the bregma using a Hamilton syringe (10 μL) fitted to a 27 G needle. The injection was delivered over a period of 3–5 min. The opening in the skull was sealed with bone wax and the skin was closed using 4–0 silk sutures with a FS-2 needle (Ethicon, Somerville NJ).

### Brain leukocyte isolation and flow cytometry analysis

Leukocytes were isolated from the brains of MCMV-infected C57B/6 WT, PD-L1 KO, and PD-1 KO mice, using a previously described procedure with minor modifications [25–28]. In brief, whole brain tissues were harvested (*n* = 3–4 animals/group/experiment) and minced finely using a scalpel in RPMI 1640 (2 g/L D-glucose and 10 mM HEPES) and digested in 0.0625% trypsin (in Ca/Mg-free HBSS) at room temperature for 20 min. Single-cell preparations of infected brains were resuspended in 30% Percoll (Sigma-Aldrich) and banded on a 70% Percoll cushion at 900 × g for 30 min at 15 °C. Brain leukocytes obtained from the 30–70% Percoll interface were collected.

Following preparation of single-cell suspensions, cells were treated with Fc block (anti-CD32/CD16 in the form of 2.4G2 hybridoma culture supernatant with 2% normal rat and 2% normal mouse serum) to inhibit non-specific Ab binding. Cells were then counted using the trypan blue dye exclusion method, and 1 × 10 [6] cells were subsequently stained with anti-mouse immune cell surface markers for 15–20 min at 4 °C (anti-CD45-PE-Cy5, anti-KLRG1-PE-Cy7, anti-CD11b-AF700, anti-CD103-PE, anti-CD127-APC, anti-CD69-e-F 450, anti-CXCR3-FITC, CCR7-PE-Cy7, anti-PD1-FITC (eBioscience, San Diego CA), and anti-CD8-BV-510 from (Biolegend)). For intracellular staining of Ki67 and Bcl-2, anti-Ki67FITC was obtained from eBioscience whereas PE-conjugated anti-Bcl-2(3F11) and PE-conjugated anti-TNP (isotype-matched control antibody for staining with anti-Bcl-2 A19-3) were from BD Pharmingen. Control isotype Abs were used for all fluorochrome combinations to assess non-specific Ab binding. Live leukocytes were gated using forward scatter and side scatter parameters on a BD FACSCanto flow cytometer and LSRII H4760 (BD Biosciences, San Jose, CA). Data were analyzed using FlowJo software (FlowJo, Ashland, OR).

### Immunohistochemistry

The brains were harvested from infected mice that were perfused with serial washes of phosphate-buffered saline (PBS), 2% sodium nitrate to remove contaminating blood cells, and 4% paraformaldehyde. The murine brains were subsequently submerged in 4% paraformaldehyde for 24 h and transferred to 25% sucrose solution for 2 days prior to sectioning. After blocking (1× PBS, 10% normal goat serum, and 0.3% Triton X-100) for 1 h at room temperature, brain sections (25 μm) were incubated overnight at 4 °C with the following primary antibodies: Rat anti-mouse CD8 (10 μg/mL; eBioscience) and Armenian Hamster anti-mouse CD103 (10 μg/mL; eBioscience). Brain sections were washed three times with PBS. After washing, secondary antibody (Goat anti-Rat FITC conjugate and Goat anti-Aremenian Hamster-conjugate Cy3) was added for 1 h at RT followed by nuclear labeling with Hoechst 33342 (1 μg/mL; Chemicon, Temecula, CA) and viewing under a fluorescent microscope.

### Real-time PCR

Total DNA was extracted from murine brain tissues using the QIAamp DNA mini kit (Qiagen, Valencia, CA). Total RNA was extracted from murine brain tissues using the TRIzol reagent (Invitrogen, Carlsbad, CA), treated with DNase and reverse transcribed to cDNA with oligo (dT)_12–18_, random hexmer, dNTPs (Gene Link, Hawthorne, NY), RNase inhibitor, and SuperScript™ III reverse transcriptase (Invitrogen). Mixtures of DNA or diluted cDNA, primers, and SYBR® Advantage® qPCR premix (ClonTech, Mountain View, CA) were subjected to real-time PCR (Stratagene, now Agilent Technologies, La Jolla, CA) according to the manufacturer’s protocol. Primer sequences were sense 5′- ATCTGAAACAGCCGTATATCATCTTG-3′ and antisense 5′- TCAGCCATCAACTCTGCTACCAAC-3′ for MCMV IE1 (100 bp), and sense 5′- TGCTCGAGATGTCATGAAGG-3′ and antisense 5′- AATCCAGCAGGTCAGCAAAG-3′ for HPRT (hypoxanthine phosphoribosyltransferase, 95 bp). The PCR conditions for the Mx3000P QPCR System were: 1 denaturation cycle at 95 °C for 10 s; 40 amplification cycles of 95 °C for 10 s, 60 °C annealing for 10 s, and elongation at 72 °C for 10 s; followed by 1 dissociation cycle. The relative product levels were quantified using the 2^−∆∆Ct^ method [29] and were normalized to the housekeeping gene HPRT.

### Statistical analysis

For comparing groups, two-tailed unpaired Student’s *T* test for samples was applied; *p* values ≤0.05 were considered significant.

## Results

### Reduced numbers of bT_RM_ cells in PD-L1 KO animals

**Fig. 1:**
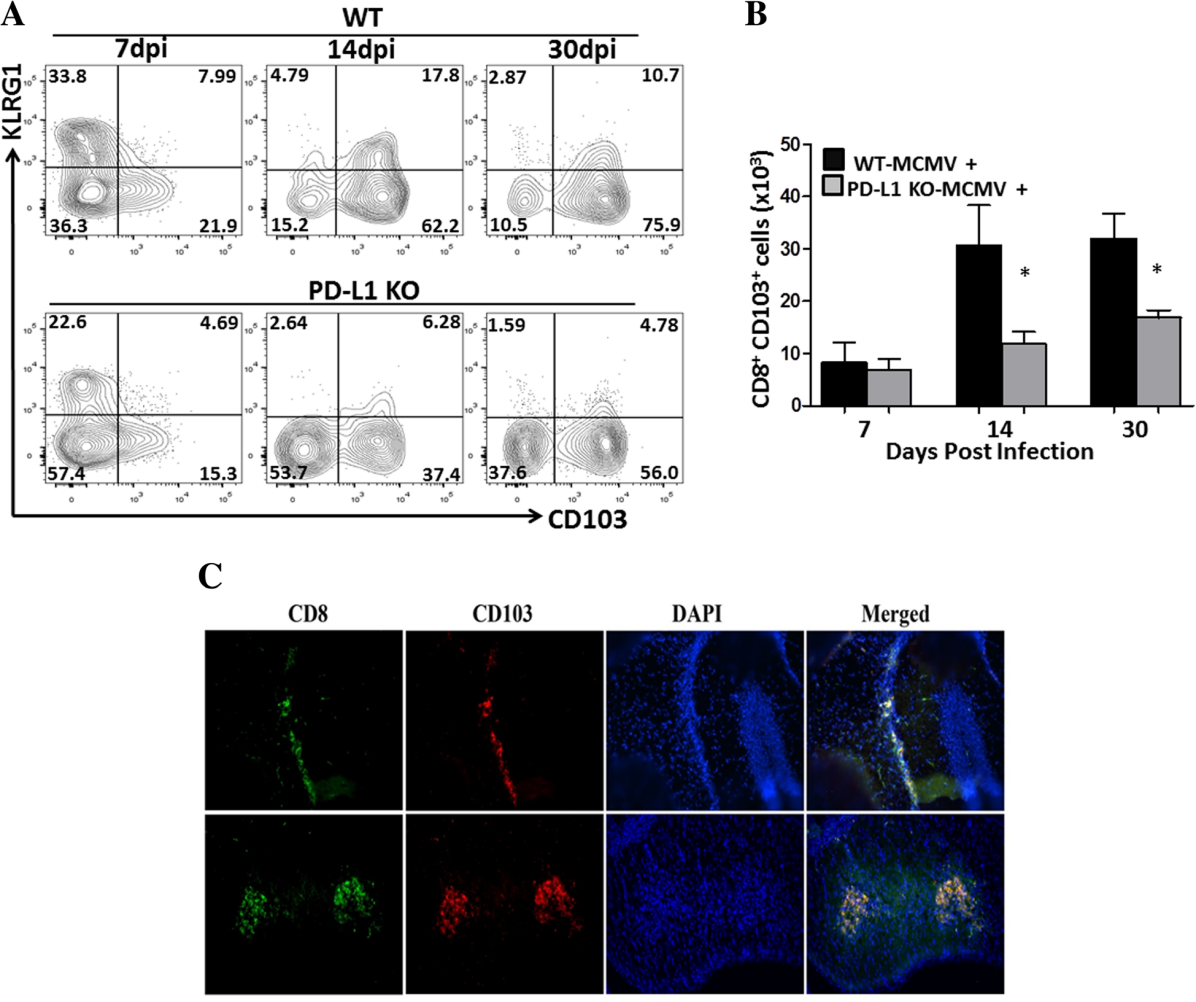
Reduced numbers of bT_RM_ cells in PD-L1 KO animals. Single-cell suspensions of brain tissue obtained from MCMV-infected WT and PD-L1 KO animals were banded on a 70% percoll cushion. Brain leukocytes were labeled with Abs specific for anti-CD45-PE-Cy5, anti-KLRG1-PE-Cy7, anti-CD103-PE, and anti-CD8-BV-510 at 7, 14, and 30 dpi and analyzed using flow cytometry. CNS-derived lymphocytes were gated on CD8^+^ T cells and analyzed for KLRG1 and CD103 (a marker for tissue-resident memory T cells in the CNS). **a** Representative *contour plots* show expression profile of KLRG1 and CD103 at the indicated time points. **b** Pooled data present absolute number (mean ± SD) of CD103^+^ CD8^+^ T cells within the infected brains of WT and PD-L1 KO animals at indicated time points from two independent experiments using three animals per group/time points. *p < 0.01 WT versus PD-L1 KO at 14 and 30 dpi. **c** Immunohistochemical staining shows the distribution of tissue-resident memory cells in the hippocampus (*upper panels*) and brain parenchyma (*lower panels*) of MCMV-infected animals at 30 dpi (magnification 20×)

In this study, we evaluated for the role of PD-1: PD-L1 signaling in the establishment of T_RM_ cells within the brain following viral infection. Kinetic studies of brain-infiltrating CD8^+^ T cells using WT and PD-L1 KO animals revealed that KLRG1^+^ cells were present within the brain during the acute phase of infection. In sharp contrast, negligible CD8^+^ T cell KLRG1 expression was noted at 30 dpi in either WT or PD-L1 KO animals. We further characterized these infiltrating KLRG1^+^ CD8^+^ T cells by assessing the expression of CD103 at various time points p.i. A progressive increase in the frequencies of the CD103-expressing population, along with concomitant lower levels of KLRG1, was observed from 7 to 30 dpi (Fig. 1a). It was noted that PD-L1 KO animals had significantly reduced numbers of CD103^+^CD8^+^ T cells within the MCMV-infected brain when compared to WT animals. This difference was found to be statistically significant at d 14 and 30 p.i. (Fig. 1b). Additionally, we collected the MCMV-infected brain from WT animals and performed immunohistochemistry staining to visualize the location of T_RM_ cells at 30 dpi, the time point of maximal CD103 expression detected by flow cytometry. These immunohistochemical studies provided evidence that the majority of CD103^+^CD8^+^ T cells were located within the infected brain parenchyma, with some cells also localized to the hippocampus (Fig. 1c). We also examined viral load within the brain between WT and PD-L1 KO animals. Productive phase MCMV IE1 transcripts occurred during acute viral infection, but not within latently infected brains, at similar levels in WT and PD-L1-KO. IE1 expression was evident at 7 dpi, whereas reduced expression was observed at 30 dpi in both groups. Additionally, CNS viral DNA load was also found to be similar between the groups of animals (Additional file 1: Figure S2).

### PD-L1 supports development of long-lived memory cells following MCMV infection

**Fig. 2:**
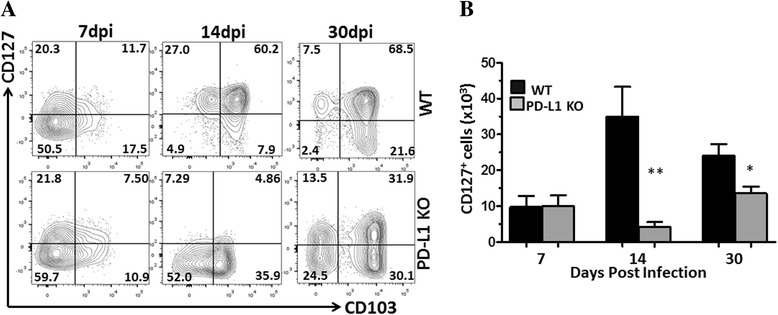
PD-L1 supports development of long-lived memory cells following MCMV infection. Brain mononuclear cells were harvested from infected WT and PD-L1 KO mice and were analyzed for expression of CD127 at the indicated time points (three animals per group per time point from two separate experiments). **a** Representative *contour plots* depict the percentage of CD127-positive cells, along with CD103^+^ expression gated on CD8^+^ T cells. **b** Data show absolute number (mean ± SD) of CD127^+^ cells within the infiltrating lymphocyte population pooled from two independent experiments. **p < 0.001 WT versus PD-L1 KO at 14 dpi and *p < 0.01 WT versus PD-L1 KO at 30 dpi

To investigate the role of PD-1: PD-L1 signaling in the development of T cell memory within the brain, we analyzed the expression profile of the interleukin (IL)-7 receptor (i.e., CD127) on these cells from 7 to 30 dpi. These studies showed that among WT animals, expression of CD127 increased as infection progressed from acute into chronic phases (Fig. 2a). In contrast, PD-L1 KO animals displayed significantly reduced long-lived memory cells, as assessed through expression of CD127 at 14 and 30 dpi, when compared to WT animals (Fig. 2b).

### bT_RM_ cells are also reduced in PD-1 KO animals

**Fig. 3:**
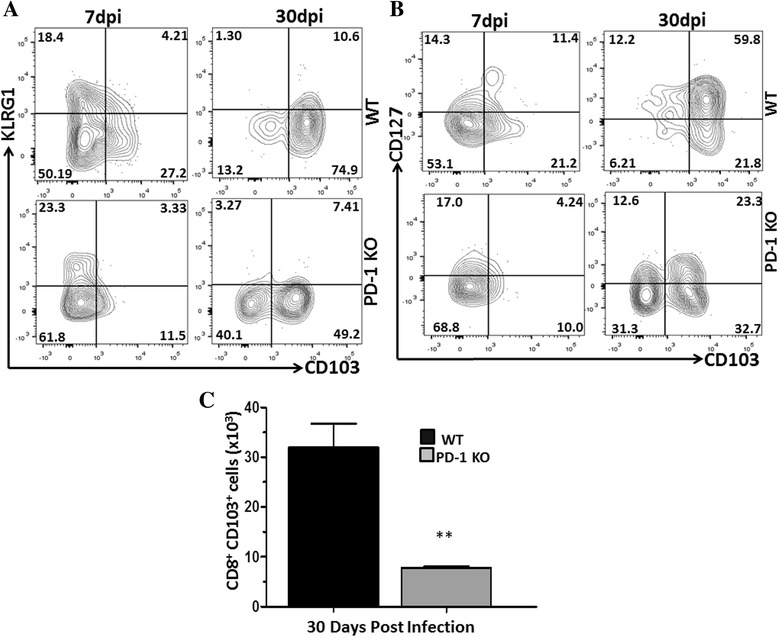
The PD-1: PD-L1 pathway promotes establishment of bT_RM_ cells following MCMV infection. Infected WT and PD-1 KO mice were sacrificed and brain mononuclear cells were collected at indicated time points. Flow cytometric analysis of CD8-gated T cells illustrates the establishment of bT_RM_. **a** Representative *contour plots* show the expression pattern of KLRG1 along with the percentage of CD103^+^ T cells within the infected brains of WT and PD-1 KO mice at 7 and 30 dpi. **b** Representative flow cytometric analysis of CD127 expression on gated CD8^+^ T cells at the indicated time points. **c** Pooled data show the number (mean ± SD) of T_RM_ cells within the brain of WT and PD-1 KO animals at 30 dpi from two independent experiments using two to three animals per group/time points. **p < 0.001 WT versus PD-1 KO at 30 dpi

Having found reduced numbers of bT_RM_ cells within the brains of PD-L1 KO mice, we went on to evaluate the expression of KLRG1, CD127, and CD103 in PD-1 KO animals to confirm these data. As seen with PD-L1 KOs, examination of these distinct surface markers by flow cytometry revealed striking differences when compared to WT animals. During the acute phase of infection (i.e., 7 dpi), KLRG1 expression, which decreased by 30 dpi, was observed in both PD-1 KO and WT animals (Fig. 3a). In contrast, expression of CD127 was significantly reduced in PD-1 KO animals when compared to WT at 30 dpi (Fig. 3b). We went on to characterize bT_RM_ in PD-1 KO animals for expression of CD103. These studies showed decreased expression of CD103 on CD8^+^ T cells isolated from the brains of PD-1 KO animals (Fig. 3a, b). We also determined the number of CD103^+^CD8^+^ T cells in PD-1 KO versus WT animals. As was the case in PD-L1 KOs, we observed fewer CD103^+^CD8^+^ T cells when compared to WT animals (Fig. 3c). These observations support the contribution of PD-1: PD-L1 signaling in the development of long-lived memory cells within the MCMV-infected brain.

### Loss of PD-L1 or PD-1 resulted in fewer CD8^+^ T cells which co-express CD69 and CD103

**Fig. 4:**
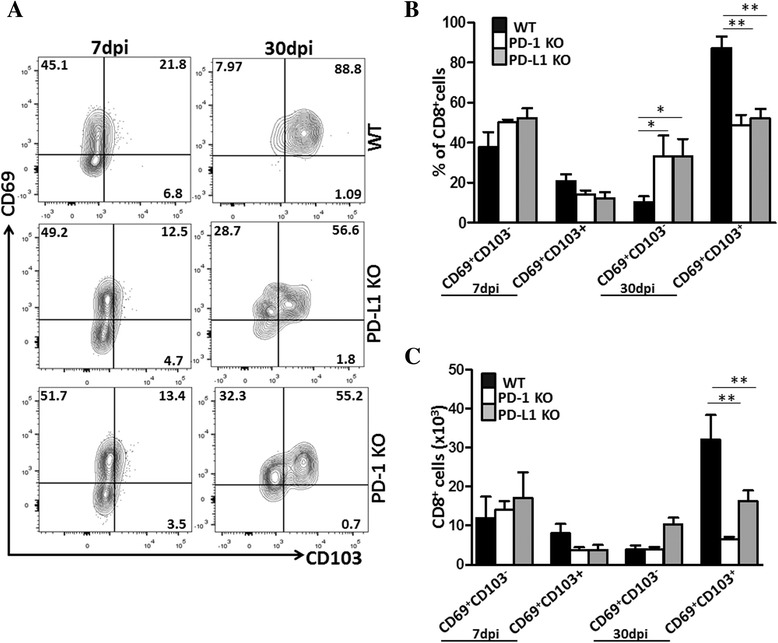
Loss of PD-L1 or PD-1 resulted in fewer CD8^+^ T cells which co-express CD69 and CD103. Brain mononuclear cells from MCMV-infected WT, PD-L1 KO, and PD-1 KO mice were collected at 7 and 30 dpi and stained for flow cytometric analysis. CD8^+^ T cells were gated from the CD45^+^CD11b^low^ leukocyte population and analyzed for co-expression of CD69 and CD103. **a** Plots show expression of the CD69 and CD103 populations and are representative of two separate experiments using two to three animals/time point/group. **b** Pooled data present the percentage (mean ± SD) of co-expressed CD69 and CD103 cells among different groups of mice at the indicated time points. *p < 0.01 WT versus PD-1 or PD-L1﻿ KO at 30 dpi **p < 0.001 WT versus PD-L1 KO for CD69^+^CD103^+^ at 30 dpi and **p < 0.001 WT versus PD-1 KO for CD69^+^CD103^+^ at 30 dpi. **c** Absolute numbers of CD8^+^ T cells that were CD69^+^CD103^+^ and CD69^+^CD103^−^ among infected WT, PD-L1, and PD-1 KO animals at 7 and 30 dpi are shown. **p < 0.001 WT versus PD-L1-KO and PD-1 KO for CD69^+^CD103^+^ at 30 dpi

Previous studies have reported that expression of CD69 is not just a marker of activation, but it is an important regulator of immune response at sites such as the mucosa and gut [30]. So, we went on to test whether our brain-infiltrating CD8^+^CD103^+^ lymphocytes also expressed CD69. We first determined CD69 expression kinetics on brain-infiltrating CD8+ T cells. Flow cytometric analysis revealed the presence of CD69 at 7 dpi, where 20.6 ± 3.5% of the CD8^+^ T cells co-expressed CD69 and CD103 in WT animals (Fig. 4a). Frequencies of CD69^+^CD103^−^ cells were found to be high during acute infection (7 dpi), (i.e., 37.7 ± 7.4%), whereas by 30 dpi, the vast majority of CD103^+^ T_RM_ cells co-expressed CD69 (i.e., 87.3 ± 5.6%). Even though CD103^+^CD8^+^ T cells co-expressed CD69 (i.e., 52 ± 4.8% in PD-L1 KO and i.e., 48.5 ± 5.3% in PD-1 KO animals), the remaining CD69^+^CD103^−^ population was significantly higher in both the PD-L1 and PD-1 KO animals at 30 dpi (i.e., 33.1 ± 8.6% and 33.02 ± 10.4% in PD-L1 and PD-1 KO animals, respectively, versus 10.2 ± 2.8% in WT animals), (Fig. 4b). Additionally, we evaluated absolute numbers of CD69^+^CD103^+^ and CD69^+^CD103^−^ CD8^+^ T cells among infected WT, PD-L1, and PD-1 KO animals. Again, more CD69^+^CD103^+^ cells were found in the brain of WT animals in contrast to the KO mice, and the differences were found to be significant at 30 dpi (Fig. 4c). These results demonstrate the early expression of CD69 in brain-infiltrating effector CD8^+^ T cells in all animals tested. However, co-expression of CD69 and CD103, the marker of bT_RM_ cells, was highly elevated in WT mice compared to PD-1 KO and PD-L1 KO animals.

### PD-1 expression on bT_RM_ cells following MCMV infection

**Fig. 5:**
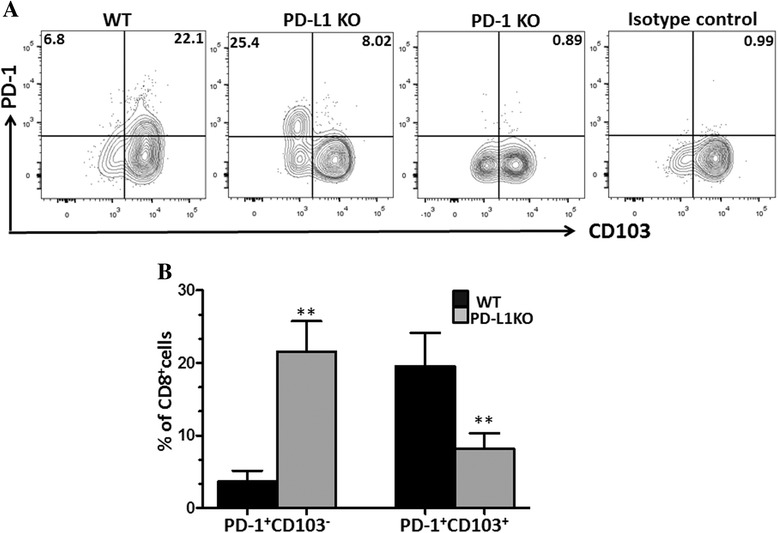
PD-1 expression on bT_RM_ cells following MCMV infection. PD-1 expression was evaluated on both CD103^+^ and CD103^−^ T cell populations within the MCMV-infected brains of WT, PD-L1 KO, and PD-1 KO mice at 30 dpi. **a** Flow cytometric plots illustrate expression of PD-1 and CD103 on CD8^+^ T cells and are representative of two separate experiments using 3–4 animals/group at 30 dpi. **b** Data are represented as mean ± SD percentage of CD8^+^ T cells which were PD-1^+^CD103^+^ and PD-1^+^CD103^−^ among WT and PD-1L KO animals at 30 dpi. **p < 0.001 WT versus PD-1 KO for PD-1^+^CD103^+^ at 30 dpi

It has been reported that expression of inhibitory receptors like CTLA-4 and PD-1 on T_RM_ cells may serve as a means to prevent these cells from unintended activation and unnecessarily attacking self [31]. To further investigate the role of the PD-1: PD-L1 pathway in the retention of resident memory cells, we went on to characterize PD-1 expression on CD103^+^CD8^+^ T cells isolated from the MCMV-infected brains at 30 dpi. In these experiments, higher frequencies of PD-1^+^CD103^+^ cells were observed within the brains of WT animals at 30 dpi when compared to PD-L1 KO mice (i.e., 19.5 ± 4.6% in WT animals versus 8.1 ± 2.1% in PD-L1 KO animals) (Fig. 5a). In contrast, an increased expression of PD-1 on CD103^−^ cells was found within the brains of PD-L1 KO animals (i.e., 21.5 ± 4.1% in PD-L1 KO animals versus 3.6 ± 1.5% in WT animals), (Fig. 5a, b). Thus, upregulation of PD-1 receptors on CD103^+^ T_RM_ cells may help preserve the longevity of T_RM_ cells.

### Expression of CXCR3 on bT_RM_ following MCMV infection

**Fig. 6:**
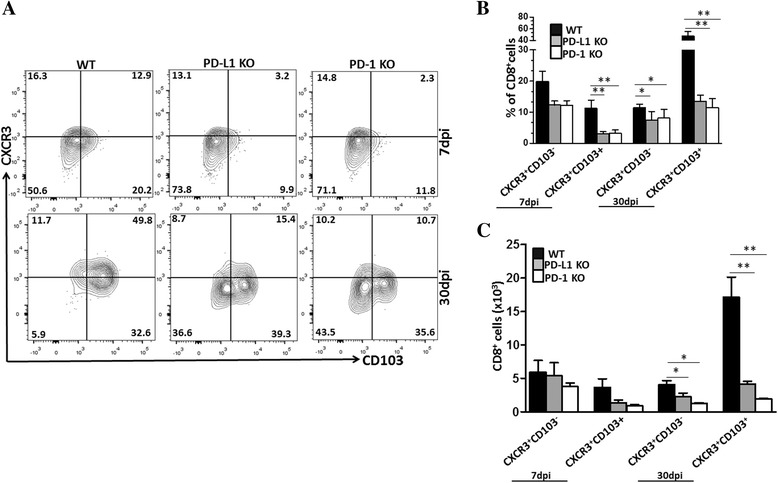
The PD-1: PD-L1 pathway promotes expression of CXCR3 on bT_RM_ following MCMV infection. Expression kinetics of the CXCR3 chemokine receptor on bT_RM_ cells. **a** Representative *contour plots* show the percentage of CXCR3 chemokine receptor expressing bT_RM_ cells, as assessed using flow cytometry at 7 and 30 dpi (*n* = 2–3 animals/group/time point from two independent experiments). **b** Data shown are mean ± SD percentage of CXCR3^+^CD103^+^ and CXCR3^+^CD103^−^ T cells among the WT, PD-L1 KO, and PD-1 KO animals at 7 and 30 dpi. *p < 0.01 WT versus PD-1 or PD-L1﻿ KO at 30 dpi **p < 0.001 WT versus PD-1 KO and WT versus PD-L1 KO for CXCR3^+^CD103^+^ expression at 30 dpi. **c** Pooled data represent absolute numbers of CD8^+^ T cells that were CXCR3^+^CD103^+^ and CXCR3^+^CD103^−^ among infected WT, PD-L1, and PD-1 KO animals at 7 and 30 dpi. **p < 0.001 WT versus PD-L1-KO and PD-1 KO for CXCR3^+^CD103^+^ at 30 dpi

The chemokines CXCL9 and CXCL10 have been shown to facilitate entry into the epithelium during infection of mucosal surfaces with HSV-2 [32]. Microglial cells have been shown to produce high levels of the chemokines CXCL9 and CXCL10 in response to viral infection [33]. So, we next sought to identify the chemokine receptor CXCR3 on bT_RM_ cells, which is a ligand for both CXCL9 and CXCL10. To help understand the role of the PD-1: PD-L1 pathway in regulating chemokine receptor expression on bT_RM_ and its contribution to their retention, we analyzed the expression pattern of CXCR3 and CCR7 within the MCMV-infected brain using flow cytometry at 7 and 30 dpi. Data obtained from these studies revealed that a large fraction of CD8^+^ T cells expressed CXCR3 along with CD103 among the WT animals at 30 dpi (Fig. 6a). Interestingly, the PD-1 KO and PD-L1 KO animals displayed much reduced expression of CXCR3 on CD103^+^ cells at 30 dpi (i.e., 13.5 ± 2.1% and 11.8 ± 4.2% in PD-1 and PD-L1 KO animals, respectively, versus 47.3 ± 8.0% in WT animals); moreover, the frequency of CXCR3 was also higher (11.2 ± 2.6%) in the CD103^+^ population among the WT animals when compared to PD-1 KO (3.1 ± 1.1%) and PD-L1 KO (3.1 ± 0.6%) at 7 dpi (Fig. 6a, b), supporting its role in retention of resident memory cells. We also determined the absolute numbers of CXCR3^+^CD103^+^ and CXCR3^+^CD103^−^ CD8^+^ T cells among infected WT, PD-L1, and PD-1 KO. We observed a difference in the number of CXCR3^+^CD103^+^ cells within the brain of WT animals in comparison to the KO animals, which were found to be significant at 30 dpi (Fig. 6c). In addition, we also investigated the expression of CCR7 on bT_RM_ cells. It has been well established that CCR7 is required for T cells to exit from tissues [17, 34]. Low-level expression of CCR7 was observed in WT animals at both 7 and 30 dpi. A similar low-level expression was noted when PD-1 KO and PD-L1 KO animals were evaluated (Additional file 2: Figure S1B).

### Bcl-2 expression on CD103^+^ and CD69^+^ CD8 T cells within the infected brain

**Fig. 7:**
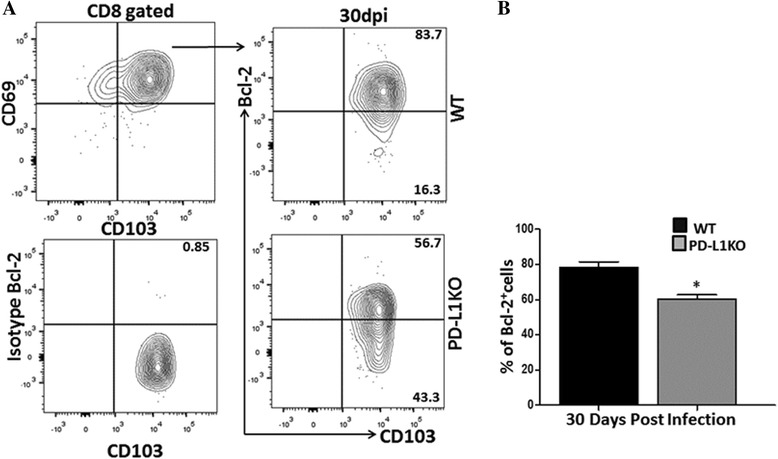
CD103^+^ and CD69^+^ CD8 T cells express Bcl-2 within the infected brain. Cells were isolated from the brains of MCMV-infected WT and PD-L1 KO mice at 30 dpi and evaluated for the expression of the pro-survival molecule Bcl-2 on CD103^+^CD69^+^-gated CD8^+^ T cells. **a** Representative flow cytometric analysis of intracellular expression of Bcl-2 expression in CD103^+^CD69^+^-gated CD8^+^ T cells among WT and PD-L1 KO animals at 30 dpi. **b** Pooled data present the percentage (mean ± SD) of Bcl-2 expression on CD103^+^CD69^+^ cells among different groups of mice at the indicated time point (*n* = 3 from both groups). *p < 0.01 WT versus PD-L1 KO for Bcl-2 expression in CD69^+^CD103^+^ cells at 30 dpi

To further analyze the retention of bT_RM_ cells and evaluate the contribution of PD-1: PD-L1 pathway in bT_RM_ generation, we analyzed expression of the pro-survival molecule Bcl-2. Levels of Bcl-2 expression were compared between WT and PD-L1 KO animals at 30 dpi. We found high expression of Bcl-2 on CD103^+^ and CD69^+^ CD8 T cells among WT animals (Fig. 7a). In contrast, PD-L1 KO animals displayed a significant decrease in Bcl-2 expression when compared with WT (78.4 ± 5.4% and 60.1 ± 4.5% in WT and PD-L1 KOs, respectively), (Fig. 7a, b). The reduction in percentage of Bcl-2 was 22.9 ± 4.5%. In addition to examining Bcl-2 expression, we also performed Ki67 staining to investigate proliferation of CD103^+^ and CD69^+^ CD8^+^ T cells among the two groups of animals. Little difference was found in the expression of Ki67 among the two groups (20.6 ± 1.1% and 17.3 ± 1.2% in WT and PD-L1 KOs, respectively), (Additional file 2: Figure S1C).

## Discussion

The most significant finding presented in this study is that the PD-1: PD-L1 pathway contributes to development of bT_RM_ cells within the MCMV-infected brains. Upon resolution of acute viral infection, the greatly expanded effector CD8^+^ T cell population rapidly contracts, leaving behind a small number of cells that survive to form long-lived memory cells [31, 35]. Some of these memory T lymphocytes persist long term in non-lymphoid tissues as T_RM_ cells, which defend against re-infection [3, 14, 36]. We and others have previously shown that effector CD8^+^ T cell populations exhibit heterogeneity in expression of KLRG1 during activation and expansion [2, 3, 7, 9]. Through study of both acute and long-term CNS viral infection using WT, PD-L1 KO, and PD-1 KO animals, we report here that brain-infiltrating CD8^+^ T cells display distinct phenotypes of SLEC and MPEC populations from acute to chronic infection. In accordance with other studies where it has been reported that CD127 and KLRG1 are inversely expressed on SLEC and MPEC, our results show that during acute MCMV infection, KLRG1^+^ CD127^−^ (i.e., the SLEC population) cells dominate. In contrast, later time points correlate with development of KLRG1^−^ CD127^+^ cells in WT animals [9, 37, 38]. Importantly, CD127 expression was significantly reduced in PD-L1 KO and PD-1 KO animals. Taken together, these data demonstrate that the PD-1: PD-L1 pathway within the CNS promotes development of a bT_RM_ cell population following viral infection.

Studies of HIV-1 infection have reported expansion of CD8^+^CD127^−^ effector-like T cells as a consequence of heightened immune responses [39]. Experiments using acute LCMV and Listeria infections in mice have demonstrated emergence of CD127-expressing CD8^+^ T cells that arise during the effector phase and acquire phenotypical and functional properties of memory T cells [37, 40]. The down-regulation of CD127 during these chronic viral infections has been attributed to ongoing repetitive TCR stimulation, whereas elevated expression of CD127 on HCV-, HBV-, and RSV-specific memory CD8^+^ T cells has been explained by a lack of persisting antigen [38, 41]. Thus, the frequency of CD127 expression on bT_RM_ cells in WT animals despite persistence of the latent viral genome may suggest an absence of ongoing TCR triggering within the MCMV-infected brain. In contrast, significantly reduced expression of CD127 on bT_RM_ cells indicates prolonged, effector-like T cell responses in PD-1 KO and PD-L1 KO animals.

Phenotypic signatures indicative of bT_RM_, consisting of CD103, CD69, and CD127 expression, were observed at higher levels among WT animals than among PD-L1 KO and PD-1 KO mice during chronic infection. Similar to findings reported for other non-lymphoid organs, as well as from the brain with vesicular stomatitis virus [42, 43], we found 87.3 ± 5.6% of the CD8^+^ T cells persisting within the MCMV-infected brain express CD69. In contrast to brain infection with LCMV, which showed that CD103 was expressed only on a portion of bT_RM_ [44], we observed that the vast majority of CD8^+^ T cells co-expressed CD103 and CD69 in WT mice during long-term infection. Expression kinetic studies show early induction of CD69 on brain-infiltrating T cells, as shown by Mutnal et al. [28], and CD69^+^CD103^−^ cells appear to show effector function early after brain infection. It has been previously reported that expression of CD69 is required for efficient effector T cell retention in the skin and subsequent formation of T_RM_ cells [11, 15, 45, 46]. This is because CD69 expression by T_RM_ cells downregulates cell surface expression of S1P1, thereby blocking T cell movement out of tissues supporting their stationary state [3, 47, 48]. In this study, PD-1 KO and PD-L1 KO animals show a dominating population of CD69^+^CD103^−^ at 30 dpi, a time point at which these mice have significantly fewer co-expressing CD69^+^ CD103^+^ cells when compared to WT. Accumulating evidence also indicates a contribution of cytokines like TGFβ, IL-15, IL-7, and IFN-α/β in the induction CD103 and CD69 [2, 49–51].

Development of T_RM_ cells in a particular tissue clearly involves various factors such as T cell migration, entry into the tissue, retention, and survival. These factors are likely regulated or induced by locally derived signals. Therefore, effector T cell populations during acute infection and the retention of T_RM_ within non-lymphoid tissue under specific environment conditions, such as the infected brain, are critical to understand. The chemokines CXCL9 and CXCL10 have been shown to facilitate entry of T cells into epithelium during infection of mucosal surfaces with HSV-2 [32]. Similarly, CXCR3, the receptor for CXCL9 and CXC10, is required for the appropriate localization of effector T cells and for subsequent formation of T_RM_ [16]. Expression of CXCR3 on circulating T cells or its chemokine ligands, CXCL9 and CXCL10, in tumor tissues has been reported to be associated with elevated intratumoral T cell infiltration in melanoma and colorectal cancer patients [52–54]. Interestingly, previous studies from our laboratory have shown that microglial cells produce high levels of CXCL9 and CXCL10 in response to MCMV brain infection [33]. Additionally, reports using a skin model suggest that CCR7 is responsible for exit of T cells out of the tissue, whereas CCR7^−^ T cells remained in the skin as T_RM_ cells [3, 11]. Likewise, results presented here show negligible expression of CCR7 at 7 and 30 dpi in all groups of animals, which are in line with other studies [3, 17]. Furthermore, differential expression of CXCR3^+^ on CD103^+^CD8^+^ T cells was observed among WT, PD-1 KO, and PD-L1 KO animals. A significantly higher level expression of CXCR3 on CD103^+^CD8^+^ T cells from the brains of WT mice in comparison to PD-1 KO and PD-L1 KO mice at 30 dpi was observed. Moreover, the PD-1: PD-L1 pathway has been reported to negatively regulate chemokine expression in various contexts. For example, increased expression of the chemokine CXCL9 and its receptor is associated with blocking of PD-L1 in dry eye disease mice [55]. Here, differential expression profiles of CXCR3 on bT_RM_ between WT, PD-1 KO, and PD-L1 KO mice were observed. This finding reveals a role for PD-1: PD-L1 in regulating the expression of CXCR3, which in-turn may regulate the retention of bT_RM_ following MCMV infection.

The PD-1: PD-L1 pathway is well known to limit immune-mediated tissue damage caused by over-reactive T cells, particularly in immune-privileged sites like the brain. Previous reports which show upregulation of PD-L1 in inflamed brain suggest a role for this pathway in regulating T cell activation, as well as controlling immunopathological damage [19, 56]. Additionally, PD-1 was first regarded as an inhibitory marker and found to be upregulated on exhausted T cells, as defined by reduced ability to proliferate and produce cytokines [57]. It has previously been proposed that increased expression of inhibitory markers, such as PD-1 and CTLA-4, on brain T_RM_ cells may serve as a means to prevent this population from unintentional activation and unnecessarily self-attack [31]. Similarly, our flow cytometry analysis reveals upregulation of PD-1 on CD103^+^CD8^+^ T cells, along with negligible expression on CD103^−^CD8^+^ cells in WT animals. Interestingly, inverse expression of PD-1 on the CD103^+^ and CD103^−^ population was observed among the PD-L1 KO animals. It appears that expression of PD-1 by bT_RM_ cells is not only a mechanism by which the immune system exerts brakes on unnecessary T cell stimulation and proliferation, but it also may itself promote longevity. Furthermore, the decreased expression of PD-1 on T_RM_ cells in PD-L1 KO animals indicates a dysregulation of bT_RM_ cells in the absence of the PD-1: PD-L1 pathway.

Evaluation of Bcl-2 expression in memory cells during chronic infection showed significant higher levels of this pro-survival factor in CD103^+^ and CD69^+^ CD8 T cells among WT when compared to PD-L1 KO animals. However, an evaluation of the proliferation potential of memory cells using Ki67 staining revealed no major difference among the two groups of animals. These data indicate increased survival of memory cells without any change in proliferation with an intact PD-1: PD-L1 pathway. Our finding was similar to Grayson et al. who reported surviving memory cells contain higher levels of Bcl-2 than naïve cells. These elevated levels of Bcl-2 may lead to diminished death phase after secondary infection, resulting in a net increase in memory cells [58].

Data indicate that T_RM_ cells residing in a variety of tissues accelerate and improve clearance of pathogens upon re-challenge. However, the driving mechanisms still remain the subject of intense investigation [31, 59]. It has recently been reported that T_RM_ cells respond to viral reactivation by the production of inflammatory cytokines, such as IFN-γ, along with immune cells being rapidly recruited from the circulation [60]. The function of T_RM_ cells in the brain may largely depend upon rapid IFN-γ production in combination with release of cytotoxic granules and perforin because IFN-γ-deficient bT_RM_ fails to provide sufficient non-cytolytic antiviral function [44]. The positioning of bT_RM_ within the brain parenchyma could be critical to rapidly eliminate infected cells in response to reinfection or reactivation of latent CNS infections.

## Conclusions

Taken together, our results indicate that bT_RMs_ are present within the CNS following viral infection and the PD-1: PD-L1 pathway plays a role in the generation of this brain-resident population.

## Additional files

Additional file 1: Figure S2. Similar viral loads within the brains of WT and PD-L1 KO animals. A. Brain tissue was isolated from MCMV-infected WT and PD-L1 KO mice at 7, 14, and 30 dpi, and extracted RNA was used to assess expression of the MCMV immediate-early IE1 gene using real-time PCR during acute and latent phases of infection. B. Viral DNA load in WT and PD-L1 KO animals was assessed using primers specific for viral IE1 genomic regions at the indicated time points. (TIF 111 kb)

Additional file 2: Figure S1. Decreased expression of CCR7 within the MCMV-infected brain. Mononuclear cells were isolated from the brains of MCMV-infected WT, PD-L1 KO, and PD-1 KO mice at 7 and 30 dpi and used for flow cytometric analysis of CCR7 expression. A. Gating strategy used for analysis of brain-derived leukocytes. B. Representative contour plots show the percentage of brain-infiltrating CD8^+^ T cells expressing CCR7 at the indicated time points. C. Representative contour plots show the percentage of Ki67^+^ cells on CD103^+^ CD8^+^-gated T cells at 30 dpi. (TIF 238 kb)

## Abbreviations

Bcl-2: B cell lymphoma 2
bT_RM_: Brain-resident memory T cells
CNS: Central nervous system
dpi: Day post-infection
KLRG1: Killer cell lectin-like receptor G1
MCMV: Murine cytomegalovirus
MPEC: Memory precursor effector cells
SLEC: Short-lived effector cells
WT: Wild type
