# Supplementary figures and images for: The PD-1: PD-L1 pathway promotes development of brain-resident memory T cells following acute viral encephalitis
Source: J Neuroinflammation. 2017 Apr 13;14:82. doi: 10.1186/s12974-017-0860-3 (PMC5390367; doi:10.1186/s12974-017-0860-3)

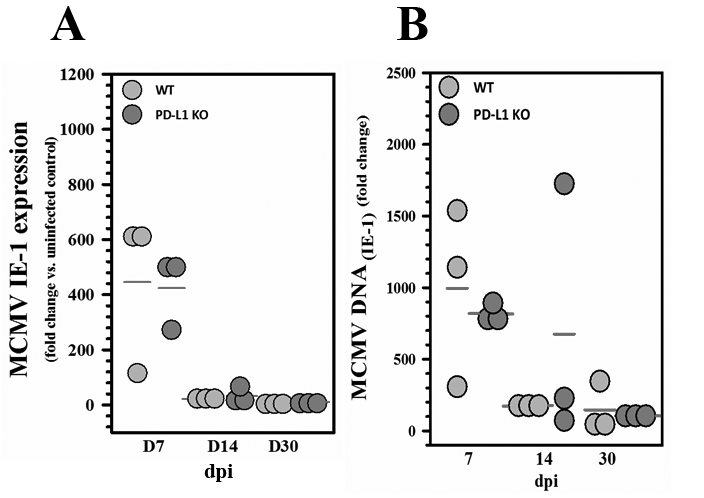

Supplement: Supplementary file 1 — Similar viral loads within the brains of WT and PD-L1 KO animals. A. Brain tissue was isolated from MCMV-infected WT and PD-L1 KO mice at 7, 14, and 30 dpi, and extracted RNA was used to assess expression of the MCMV immediate-early IE1 gene using real-time PCR during acute and latent phases of infection. B. Viral DNA load in WT and PD-L1 KO animals was assessed using primers specific for viral IE1 genomic regions at the indicated time points. (TIF 111 kb) [file 12974_2017_860_MOESM1_ESM.tif]

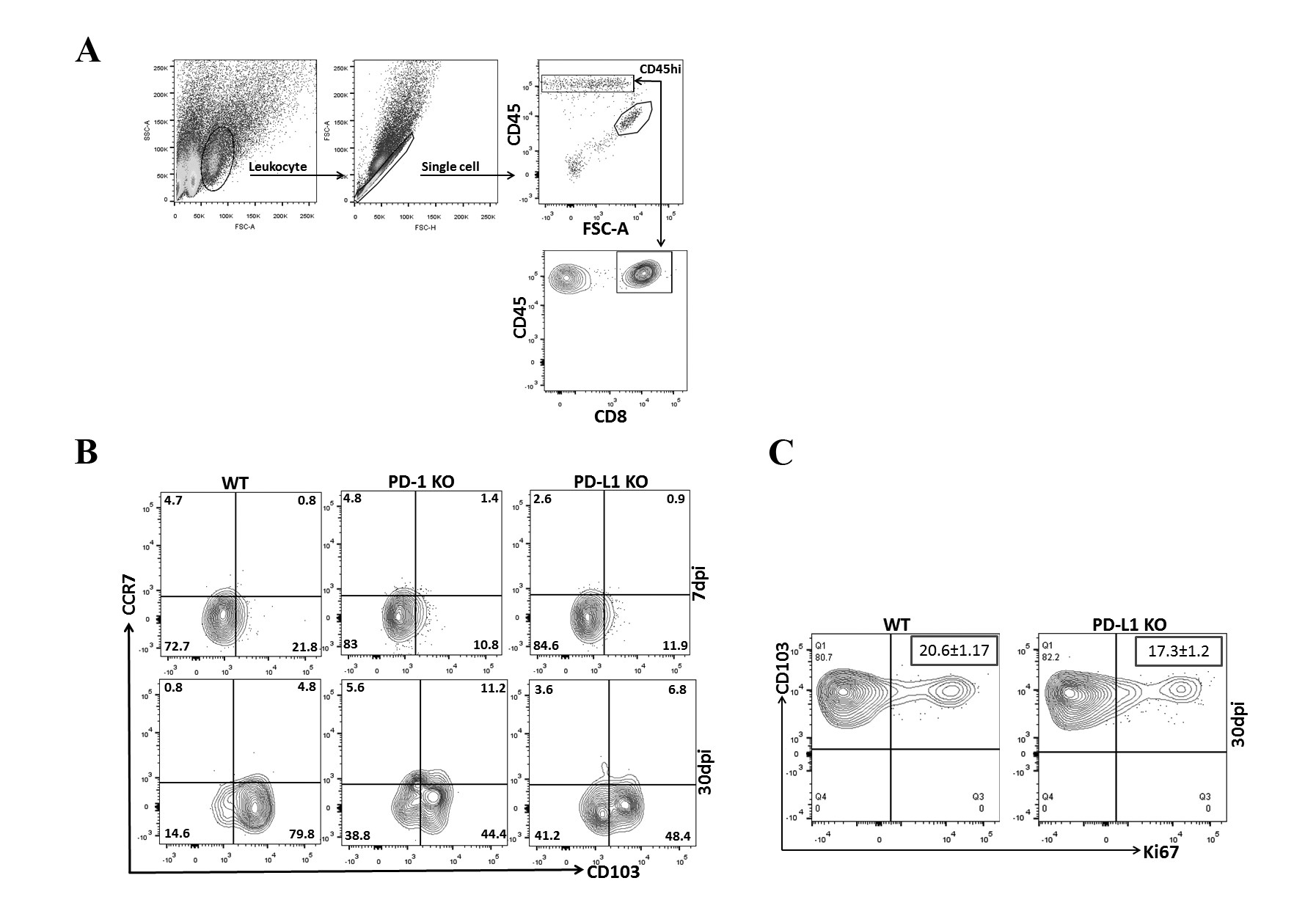

Supplement: Supplementary file 2 — Decreased expression of CCR7 within the MCMV-infected brain. Mononuclear cells were isolated from the brains of MCMV-infected WT, PD-L1 KO, and PD-1 KO mice at 7 and 30 dpi and used for flow cytometric analysis of CCR7 expression. A. Gating strategy used for analysis of brain-derived leukocytes. B. Representative contour plots show the percentage of brain-infiltrating CD8+ T cells expressing CCR7 at the indicated time points. C. Representative contour plots show the percentage of Ki67+ cells on CD103+ CD8+-gated T cells at 30 dpi. (TIF 238 kb) [file 12974_2017_860_MOESM2_ESM.tif]
